# Antioxidant and Antihypertensive Effects of a Chemically Defined Fraction of Syrah Red Wine on Spontaneously Hypertensive Rats

**DOI:** 10.3390/nu9060574

**Published:** 2017-06-03

**Authors:** Eugênia Abrantes de Figueiredo, Naiane Ferraz Bandeira Alves, Matheus Morais de Oliveira Monteiro, Clenia de Oliveira Cavalcanti, Tania Maria Sarmento da Silva, Telma Maria Guedes da Silva, Valdir de Andrade Braga, Eduardo de Jesus Oliveira

**Affiliations:** 1 Centro de Biotecnologia, Programa de Pós Graduação em Produtos Naturais e Sintéticos Bioativos, Campus I, João Pessoa, PB 58051-970, Brazil; eugeniafigueiredo.farmacia@gmail.com (E.A.d.F.); naiferraz@gmail.com (N.F.B.A.); monteirommo@gmail.com (M.M.d.O.M.); cleniacavalcanti91@gmail.com (C.d.O.C.); valdir@cbiotec.ufpb.br (V.d.A.B.); 2 Departamento de Química, Laboratório de Bioprospecção Fitoquímica, Universidade Federal Rural de Pernambuco, Recife, PE 52171-900, Brazil; sarmento.silva@gmail.com (T.M.S.d.S.); guedes.meira@gmail.com (T.M.G.d.S.); 3 Departamento de Farmácia, Universidade Federal dos Vales do Jequitinhonha e Mucurí, Diamantina, MG 39100-000, Brazil

**Keywords:** red wine, phenolics, hypertension, flavonoids

## Abstract

A particularly phenolic-rich fraction extracted from red wine from the São Francisco valley (Northeastern Brazil) was chemically characterized and its hypotensive and antioxidant effects on spontaneously hypertensive rats were studied both in vitro and in vivo. The liquid-liquid pH dependent fractionation scheme afforded a fraction with high content of bioactive phenolics such as flavonols, flavonol glycosides, phenolic acids and anthocyanins, whose identities were confirmed by liquid chromatography coupled to mass spectrometry analysis. Pretreatment of spontaneously hypertensive rats with this wine fraction at doses of 50 and 100 mg/kg by gavage for 15 days was able to decrease mean arterial pressure and heart rate as well as decrease serum lipid peroxidation. The fraction at concentrations of 0.01–1000 µg/mL induced concentration-dependent relaxation of isolated rat superior mesenteric artery rings pre-contracted with phenylephrine and this effect was not attenuated by endothelium removal. Our results demonstrate it is possible for phenolic constituents of red wine that are orally bioavailable to exert in vivo hypotensive and antioxidant effects on intact endothelial function.

## 1. Introduction

Red wine is the result of grape fermentation processes, and is characterized by high levels of polyphenols, including flavonoids, such as flavonols, flavones, proanthocyanidins, anthocyanins, and catechins; and non-flavonoid compounds which include derivatives of hydroxycinnamic acid, benzoic acid, hydrolysable acids and stilbenes, such as resveratrol [1,2].

Climactic factors such as temperature, humidity and solar radiation exert great influence on the development, production and quality of grapes and therefore wine, as well as their phenolic compound contents. Located in the Northeastern region of Brazil between the states of Pernambuco and Bahia, at latitude 8 to 9° S and longitude 40° W, the São Francisco Valley (SFV) is a recently developed wine-producing region in the country. This area has a tropical semi-arid climate with high temperatures, high brightness, abundant water for irrigation, sand-clay ground and annual rainfall of 300–800 mm [3]. These climactic characteristics allow producers to harvest twice a year and red wines from the region are marked by high levels of bioactive phenolic compounds [4]. Previous studies by our group with red wines from this region have demonstrated that these wines present considerably higher levels of phenolic compounds than those reported in the literature for red wines in general [4].

The mechanisms involved in the pathophysiology of arterial hypertension are complex but an increasing body of evidence mainly accumulated in the last decade by us and others suggests the participation of reactive oxygen species (ROS) in the development and maintenance of high blood pressure [5,6,7]. Oxidative stress is involved in the development of hypertension through different mechanisms [8,9,10,11,12]. These mechanisms include for example the inactivation of nitric oxide by superoxide, leading to endothelium dysfunction and vasoconstriction [13] and the effect of oxidative stress on the oxidation of low density lipoproteins [14].

Several studies have demonstrated an association between the consumption of food and/or beverages rich in phenolic compounds and a reduction on the risk of cardiovascular diseases [15,16,17]. A large number of existing epidemiological studies showed an inverse association between consumption of a number of phenolic compounds and risk of hypertension. This evidence comes not only from cross-sectional studies [18,19,20] but also from prospective ones [21,22]. The largest of these prospective studies [21] involved 156,957 subjects with a follow-up period of 14 years and revealed an 8% reduction in the risk of developing hypertension for those in the highest quintile of anthocyanin intake when compared with those in the lowest quintile. A similar reduction of 10% in the risk of developing hypertension for those in the highest quintile of flavonoid intake compared with those in the lowest quintile was seen in a prospective cohort of 40,574 French women with a follow-up period of 16 years [22]. Experimental evidence has implicated phenolic compounds in several different mechanisms relevant in the pathogenesis of hypertension, including the lowering of platelet aggregation [23], a decrease in the oxidation of low density lipoprotein [24], and an increase of endothelium nitric oxide [25].

Previous results with the high levels of phenolics in SFV wines prompted us to prepare a fraction from the Syrah red wine of SFV that concentrates biorelevant phenolic compounds (Fr 2 SySFV), to chemically characterize this fraction and study the antioxidant and antihypertensive activity in a variety of in vitro and in vivo assays using spontaneously hypertensive rats.

## 2. Materials and Methods

### 2.1. Standards and Reagents

Gallic acid, Folin–ciocalteu reagent, 1,1-diphenyl-2-picrylhydrazyl radical (DPPH), 2,20-azino-bis-3-ethylbenzothiazoline-6-sulphonic acid (ABTS), 6-hydroxy-2,5,7,8-tetramethylchroman-2-carboxylic acid (Trolox), potassium persulphate, quercetin, *trans*-resveratrol, ascorbic acid, phenylephrine (Phe), and acetylcholine chloride, were purchased from Sigma–Aldrich (St. Louis, MO, USA). Ethyl acetate and HPLC-grade acetonitrile was from Tedia (Tedia, Brazil). Water was purified through a Milliq1 water purification system (Millipore, Billerica, MA, USA) and the other solvents were all reagent-grade.

### 2.2. Wine Sample and Fractionation

The Fr 2 SySFV was obtained from a red wine produced in the São Francisco Valley region (Syrah variety, Rendeiras winery, harvest 2013). The Syrah variety was chosen because it displayed the highest phenolic content amongst different wines from SFV, Southern Brazil (Serra Gaucha) and Chile that were screened (results not shown). Liquid–liquid extraction methods according to Ghiselli et al. (1998) [26] were used to obtain several fractions containing different classes of polyphenolic compounds. Alcohol removal was performed by vacuum distillation (50 °C). In brief, the de-alcoholized wine (100 mL) had its pH adjusted to 2.0 and was first extracted with ethyl acetate (three times with 100 mL of EtOAc each). The aqueous phase was combined and concentrated under reduced pressure with ethanol addition (at 50 °C) until dryness, producing Fr 1 SySFV. The organic phase was concentrated to dryness, redissolved in water (100 mL), had its pH adjusted to 7.0 and was then further extracted with EtOAc (three times with 100 mL of EtOAc each). The combined organic phase from this second extraction was concentrated under reduced pressure (at 50 °C) until dryness to afford Fr 2 SySFV. The aqueous residue from this extraction was adjusted to pH 2.0 and extracted again with EtOAc (three times with 100 mL of EtOAc each). The resulting organic phase from this third extraction was combined and concentrated under reduced pressure (at 50 °C) to dryness, producing Fr 3 SySFV. The aqueous phase from the third extraction was discarded.

### 2.3. Total Phenolic Content

The total phenolic content of the fractions was determined by Folin-Ciocalteu reagent according to Waterhouse (2003) [27], with gallic acid as a standard, and expressed as milligrams of gallic acid equivalents/100 milligrams of fraction (mg GAE/100 mg). Briefly, a solution at 1 mg/mL of each wine fraction was transferred to a 5 mL volumetric flask together with 100 mL of the Folin-Ciocalteu reagent and 3 mL of Milliq^®^ water; it was then agitated for 30 s. The volume was completed with Milliq^®^ water and after 2 h the absorbance of the solution was measured at 760 nm in a ultraviolet/visible (UV/Vis) spectrophotometer. Solutions of gallic acid (ranging from 0.5 to 50 mg/mL) were analyzed in a similar manner to that described above and used to construct a calibration curve. Each sample (wine fraction) was analyzed in triplicate and the total phenolic content was expressed as milligrams of gallic acid equivalents/100 milligrams of fraction (mg GAE/100 mg).

### 2.4. Antioxidant Assays

The DPPH assay was used to measure the free radical-scavenging capacity of the wine fractions, according to a previously reported method [28]. After preliminary screening, solutions were prepared in methanol at final concentrations ranging from 3 to 30 μg/mL. Subsequently, 100 μL of the samples were transferred to 96-well plates and then 100 μL of the DPPH solution (118.2 μg/mL in MeOH) was added. The solutions were shaken and after 30 min of reaction at ambient temperature the absorbance of the samples was measured on a UV/Vis spectrophotometer at 517 nm. Each sample was tested in triplicate. The percentage radical-scavenging activity (%SA) was calculated using Equation (1) below:

%SA = ((*Abs_control_* − *Abs_sample_*) × 100)/*Abs_control_*, (1)

where *Abs_control_* is the absorbance of a solution with DPPH and methanol alone, and *Abs_sample_* is the absorbance of the DPPH solution in the presence of the wine fractions or the standard used, i.e., ascorbic acid.

Trolox-equivalent antioxidant capacity (TEAC) and effective concentrations to sequester 50% of free radicals (EC_50_ values) for the fractions against ABTS^•+^ radical cation were determined following a previously published method [29], using 6-hydroxy 2,5,7,8-tetramethyl chroman 2-carboxylic acid (Trolox), a vitamin E water-soluble analog, as standard. Initially, the ABTS^•+^ radical cation solution was prepared by mixing 2.5 mL of a solution of ABTS (7.0 mM) with 44 μL of a solution of potassium persulfate (140.0 mM), both in distilled water. The solution was kept protected from direct light at room temperature for a period of 12–16 h before use. Then, the solution of the ABTS^•+^ radical was diluted with ethanol (approximately 1:80 *v/v*) obtaining an absorbance (A) of 0.7 ± 0.05 at the wavelength of 734 nm, using an UV/Vis spectrophotometer. The solutions of the samples were prepared in EtOH at concentrations of 0.5, 1.0 and 5.0 mg/mL. Through preliminary screening, appropriate quantities of the sample solutions and the ABTS^•+^ solution were transferred to 2-mL Eppendorf tubes and the volume was completed to 500 μL with EtOH. Sample concentrations ranged from 5 to 200 μg/mL. Trolox was used as the standard substance at concentrations of 0.5, 1.0, 2.0, 3.0, 4.0, 5.0 and 6.0 μg/mL. The solutions were shaken and, after 6 min of reaction, the absorbance of the samples and the standard were measured on a UV/Vis spectrophotometer at a wavelength of 734 nm. Each concentration was tested in triplicate. The percentage of sequestering activity (% SA) was calculated as described for DPPH scavenging activity.

The results of the antioxidant assays were expressed as EC_50_ ± standard deviation (SD). EC_50_ values for the fractions were obtained by linear regression (using the software Graphpad Prism, v. 5.0, Graphpad Software Inc., La Jolla, CA, USA) of the %SA values plotted against concentration and are expressed as µg of fraction/mL solution

### 2.5. Quantification of Trans-Resveratrol and Quercetin by HPLC-UV Analysis

The content of *trans* resveratrol and quercetin was determined in Fr 2 SySFV, the fraction with higher phenolic content and antioxidant activity. A reversed-phase chromatographic method to determine *trans*-resveratrol and quercetin has been previously described and validated [4]. The HPLC analyses were conducted on a Shimadzu liquid chromatograph system (Shimadzu Corp, Kyoto, Japan) equipped with a LC-10 ATvp pump, variable wavelength detector SPD 10AVvp, controller module SCL 10A vp, a LC-10AD vp pump, a vacuum degasser DGU-14A, and an autosampler. The analytes were separated on a Phenomenex C18 column (250 mm × 4.6 mm, 5 μm), using a gradient system of two eluents: acetonitrile and water containing 0.1% formic acid (35:65) at a flow rate of 1 mL/min. The detection wavelength was 307 nm for *trans*-resveratrol and 370 for quercetin. The injection volume was 20 μL. The concentration of each component of interest was calculated based on a calibration curve created from solutions of the *trans*-resveratrol standard at concentrations of 0.1, 0.3, 0.5, 1.0, 1.5 µg/mL and quercetin at concentrations of 0.5, 0.7, 1.0, 1.5, 2.0 µg/mL.

### 2.6. Chemical Characterization by Liquid Chromatography Coupled to Mass Spectrometry (LC–MS) Analysis

We investigated the chemical composition of the fraction with higher total phenolic content and highest antioxidant activity (Fr 2 SySFV). LC–MS analysis was performed on an ultra-performance liquid chromatograph ACQUITY UPLC H-Class (Waters Corporation, Milford, MA, USA) coupled to a quadrupole time-of-flight high-resolution mass spectrometer (Xevo G2-XS QTof, Waters, Manchester, UK) with electrospray ionization (UPLC-ESI-QTOF-HRMS). The mass spectrometer was connected to the ACQUITY UPLC system via an electrospray ionization (ESI) interface. Chromatographic separation of compounds was performed on the ACQUITY UPLC with a conditioned autosampler at 4 °C, using an Acquity BEH C18 column (50 mm × 2.1 mm i.d., 1.7-μm particle size) (Waters, Milford, MA, USA). The column temperature was maintained at 40 °C. The mobile phase consisting of water with 0.1% formic acid in water (solvent A) and acetonitrile (solvent B) was pumped at a flow rate of 0.4 mL min^−1^. The gradient elution program was as follows: 0–5 min, 5%–10% B; 5–9 min, 10%–95% B. The injection volume was 10 μL. MS analysis was performed in the negative ion mode. The scan range was from 50 to 1200 *m/z* for data acquisition. In addition, MS^E^ experiments were carried out allowing both precursor and product ion data to be acquired simultaneously in one injection. Source conditions were as follows: capillary voltage, 2.0 kV; sample cone, source temperature, 100 °C; desolvation temperature 250 °C; cone gas flow rate 20 L h^−1^; desolvation gas (N_2_) flow rate 600 L h^−1^. All analyses were performed using the lockspray probe, which ensured accuracy and reproducibility. Leucine–enkephalin (5 ng mL^−1^) was used as a standard or reference compound to calibrate the mass spectrometer during analysis and introduced using the lockspray probe at 10 μL min^−1^ for accurate mass acquisition. All the acquisition and analysis of data were controlled using Waters MassLynx v 4.1 software (Waters, Milford, MA, USA). Simultaneous detection using a photodiode array detector (DAD) was performed monitoring absorbance at wavelengths ranging from 210 to 500 nm.

### 2.7. Animals and Treatment

Thirty-two adult male spontaneously hypertensive rats rats (270–320 g) were housed in a temperature-controlled room, set to a 12:12-h light–dark cycle with free access to standard rat chow (Labina^®^, Purina, Paulinea, SP, Brazil) and water. When the animals aged 12 weeks, they were treated with a daily dose of Fr 2 SySFV (50 and 100 mg/kg, p.o. by gavage) or saline (0.9% NaCl) for fifteen days. All procedures described in the present study are in agreement with the rules set forth by the Institutional Animal Care and Use Committee of the Federal University of Paraiba (CEUA/UFPB protocol n° 0601/13).

### 2.8. Blood Pressure and Heart Rate Recordings

One day before the experiments, rats were anesthetized with ketamine and xylazine (75 and 10 mg/kg, respectively, both by intraperitoneal injection (i.p.)) and fitted with femoral venous and arterial catheters for drug injection and arterial pressure recordings, respectively. Blood pressure measurements were performed 24 h after catheter implantation as previously described [30]. Changes in blood pressure and heart rate were recorded in conscious rats using a pressure transducer (MLT0380/D, ADInstruments, Sydney, Australia) connected to a computer (Mikro-tip Blood pressure system, ADInstruments, Australia) running the LabChart software (ADInstruments, Australia).

### 2.9. Tiobarbituric Acid Reactive Species (TBARS) Assay

TBARS levels in samples were measured by a spectrophotometric assay that quantifies a chromogen produced by the reaction of thiobarbituric acid with malondialdehyde (MDA), which is the end product of lipid peroxidation, and reacts with TBA as a TBARS to produce a red colored complex with peak absorbance at 532 nm as described previously [31]. Initially, 250 µL of serum was collected from each group and stored at 37 °C for 1 h, after which 400 µL of 35% perchloric acid was added, and the mixture was centrifuged at 14,000 rpm for 20 min at 4 °C. The supernatant was removed, mixed with 400 µL of 0.6% thiobarbituric acid and incubated at 60 °C for 1 h. After cooling, the absorbance at 532 nm was measured. A standard curve was generated using 1,1,3,3-tetramethoxypropane. The results were expressed as nmol of MDA/mL of serum.

### 2.10. Vascular Reactivity Studies in Isolated Rat Superior Mesenteric Artery Rings

Rats were euthanized and the superior mesenteric artery was removed and cleaned from connective tissue and fat. Rings (1–2 mm) were obtained and whenever appropriated, the endothelium was removed by gently rubbing the intimal surface of the vessels and placed in physiological Tyrode’s solution. The Tyrode’s solution composition was (in mmol/L): 158.3 NaCl; 4.0 KCl; 2.0 CaCl_2_; 1.05 MgCl_2_; 0.42 NaH_2_PO_4_; 10.0 NaHCO_3_; 5.6 glucose, kept at 37 °C and gassed with a carbogenic mixture (95% O_2_ and 5% CO_2_) and maintained at pH 7.4. All preparations were stabilized under a resting tension of 0.75 g for 1 h. The solution was replaced every 15 min to prevent the accumulation of metabolites. Tension was recorded by a force transducer (PowerLab, ADInstruments, Australia). The presence of functional endothelium was assessed by the ability of acetylcholine (10 mM) to induce 85% relaxation of vessels pre-contracted with Phe (10 mM). Less than 10% of relaxation to acetylcholine was taken as evidence that the vessel segments were functionally denuded of endothelium.

The rings were again contracted with Phe (10 μM) and after about 30 min, increasing and cumulative concentrations of Fr 2 SySFV (0.01, 0.03, 0.1, 0.3, 1, 3, 10, 30, 100, 300, and 1000 μg/mL) were added to obtain a contraction-response curve. The maximal relaxation was calculated using as reference the maximum contracting response obtained to Phe (10 mM) when used at its highest concentration.

### 2.11. Statistical Analysis

Values were expressed as mean ± standard error of mean (SEM) unless otherwise stated. When appropriate, the data were analyzed by Student’s *t*-test or two-way ANOVA followed by Tukey’s post-test for multiple comparisons, using GraphPad Prism software (v. 5.0, GraphPad Software Inc., San Jose, CA, USA). Values of *p* < 0.05 were considered statistically significant.

## 3. Results

### 3.1. Phenolic Content and Antioxidant Activity

The fractionation of red wine using liquid-liquid extraction afforded three fractions (Fr 1 SySFV, Fr 2 SySFV and Fr 3 SySFV). The total phenolic content of these fractions as well as their antioxidant activity expresssed as EC_50_ values are shown in Table 1. The total phenolic content of the fractions ranged from 5.57 ± 0.01 to 58.45 ± 0.01 mg GAE/100 mg. The fraction with the highest phenolic content was Fr 2 SySFV with 58.45 ± 0.01 mg GAE/100 mg. This fraction was obtained at neutral pH and concentrates flavonoids, phenolic acids and flavonoid glycosides [26], compounds with known antioxidant activity. It was thus expected that Fr 2 SySFV would display the highest radical scavenging activity on antioxidant assays. The antioxidant activity against DPPH radical expressed as EC_50_ values varied from 3.4 ± 0.03 to 56,27 ± 5.50 μg/mL. Fr 2 SySFV was the most active of the three fractions tested (EC_50_ = 3.4 ± 0.03 μg/mL), while Fr 1 SySFV was the least active. Ascorbic acid had an EC_50_ value of 4.38 ± 0.07 μg/mL, thus showing that Fr 2 SySFV had radical-scavenging activities comparable to this standard. Similar results were found for the ABTS radical-scavenging assay (Table 1), with Fr 2 SySFV again displaying the highest antioxidant activity (EC_50_ = 4.65 ± 0.04 μg/mL). A strong correlation (*r^2^* = 0.9999) was obtained between the EC_50_ values of the fractions on the two radical-scavenging assays. Also, negative and strong correlations were obtained between total phenolic content of the fractions and their EC_50_ values at DPPH or ABTS radical-scavenging assays (*r^2^* = −0.8447 and *r^2^* = −0.8385, respectively). The content of trans-resveratrol and quercetin in Fr 2 SySFV as determined by HPLC was 1.11 ± 0.009 and 8.56 ± 0.078 µg/mL, respectively.

### 3.2. Chemical Characterization of Fr 2 SySFV by UPLC-ESI-QTOF-HRMS

**Figure 1:**
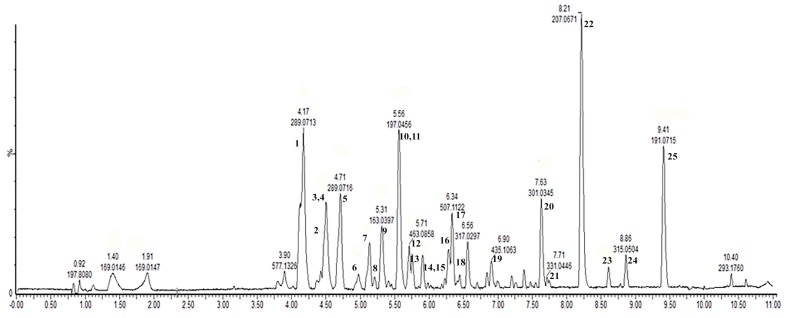
Base peak total ion chromatogram of the wine fraction (Fr 2 SySFV) obtained by an MS^E^ data collection technique method using ultra performance liquid chromatography coupled with time of flight mass spectrometry (UPLC-QTOF/MS^E^) in negative electrospray ionization mode (ESI^-^).

The chemical characterization of Fr 2 Sy SFV confirmed that this fraction concentrated flavonols, phenolic acids and flavonol glycosides. Figure 1 shows the total ion chromatogram with the marked peaks of the main compounds identified. The identification was based on comparison of the predicted versus theoretical exact mass of the compounds and also on the presence of characteristic fragment ions (Table 2) on the mass spectrum. Twenty five compounds in total were thus identified and the chemical classes included as major constituents flavonoids and its glycosides (myricetin, myricetin hexoside, dihydroquercetin hexoside, quercetin hexoside, myricetin methyl ether hexoside, dihydrokaempferol hexoside, myricetin dimethyl ether hexoside, dihydrokaempferol rhamnoside, quercetin, myricetin methyl ether, quercetin methyl ether and luteolin), but also phenolic acids and derivatives (caffeic acid, *p*-coumaric acid, syringic acid, dimethoxy-cinnamic acid, and methyl-methoxycinnamate), catechins (catechin, epicatechin, epigallocatechin-coumaroyl, and epigallocatechin-cinnamoyl) and anthocyanins (procyanidin dimer and procyanidin dimer monoglycoside). The presence of these classes of compounds in Fr 2 SySFV confirms the efficacy of the wine’s liquid-liquid fractionation scheme to concentrate the main bioactive phenolics in the neutral acetate fraction.

### 3.3. Treatment with Fr 2 SySFV Reduces Blood Pressure in Spontaneoulsy Hypertensive Rats (SHR) In Vivo

**Figure 2:**
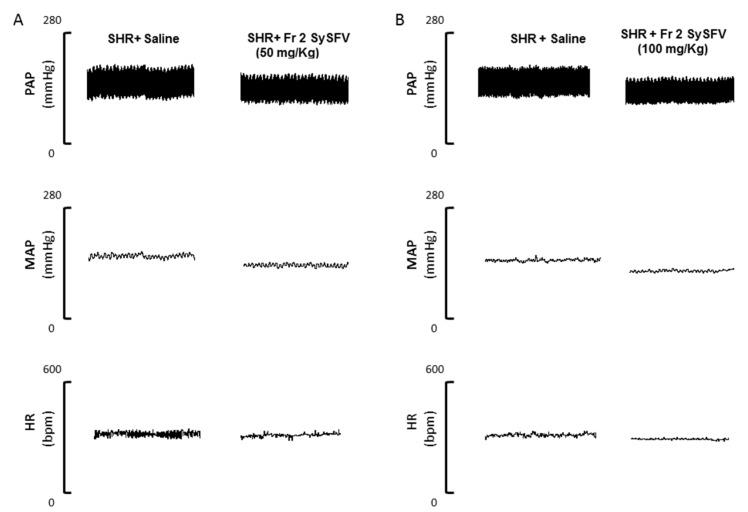
Representative tracings illustrating the changes in pulse arterial pressure (PAP, mmHg), mean arterial pressure (MAP, mmHg) and heart rate (HR, bpm) in spontaneously hypertensive rats (SHR) pretreated with Fr 2 SySFV at 50 mg/kg (**A**) and 100 mg/kg (**B**) p.o. and in saline-treated controls.

**Figure 3:**
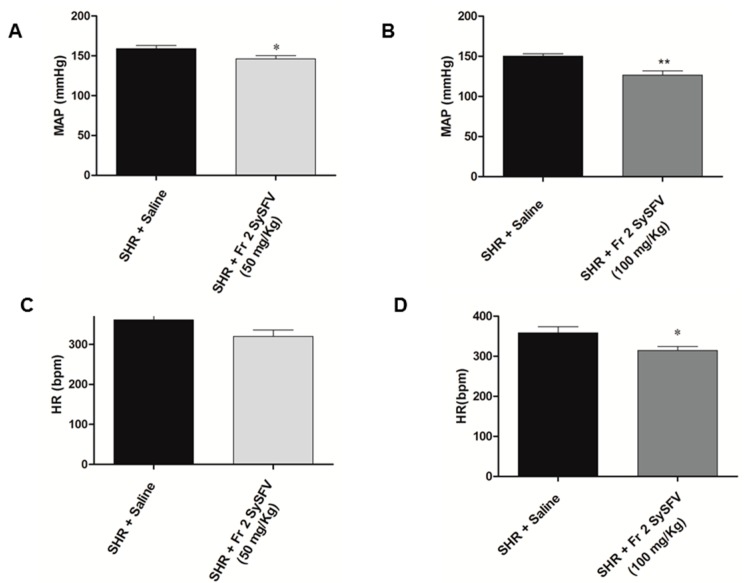
Effect of pre-treatment with Fr 2 SySFV on mean arterial pressure (MAP) of spontaneously hypertensive rats (SHR) at doses of 50 mg/kg p.o. (**A**) and 100 mg/kg p.o. (**B**) and on heart rate (HR) at 50 mg/kg p.o. (**C**) and 100 mg/kg p.o. (**D**) compared to saline-treated controls. * *p* < 0.05 and ** *p* < 0.005 when compared to SHR + saline group. Values are mean ± SEM., *n* = 7 for 50 mg/kg groups and *n* = 8 for 100 mg/kg groups

Figure 2 shows representative tracings illustrating changes in pulse arterial pressure (PAP), mean arterial pressure (MAP) and heart rate (HR) in SHR groups pre-treated with Fr 2 SySFV and saline-treated controls. Treatment of animals with Fr 2 SySFV for fifteen days at 50 mg/kg by gavage (p.o) significantly reduced blood pressure when compared to the control group (146.1 ± 4.062 *n* = 7 vs. 159.0 ± 3.891 mmHg, *n* = 7, respectively) as well as when administered at 100 mg/kg (126.5 ± 5.322 *n* = 8 vs. 150.0 ± 3.039 mmHg, *n* = 7, respectively). Only the 100 mg/kg dose was able to significantly decrease the heart rate compared to the control group (314.6 ± 9.507, *n* = 8, 358.6 ± 15.00 bpm, *n* = 7, respectively). These results are also shown in the group data in Figure 3.

### 3.4. Treatment with Fr 2 SySFV Reduces Oxidative Stress in Spontaneoulsy Hypertensive Rats

**Figure 4:**
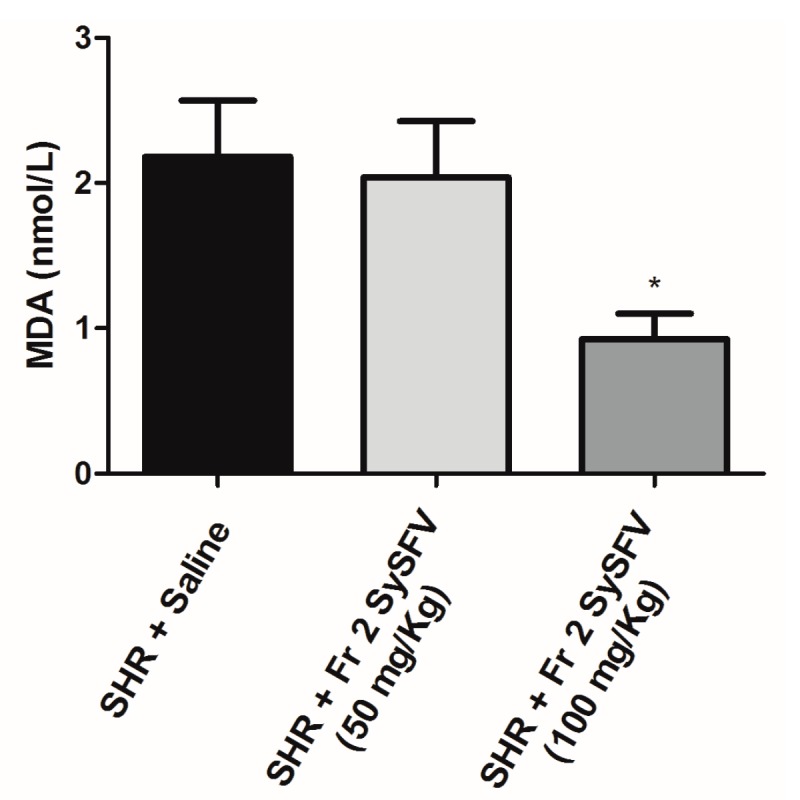
Levels of serum malondialdehyde (MDA) in spontaneously hypertensive rats pre-treated with Fr 2 SySFV for 15 days p.o. at doses of 50 mg/kg and 100 mg/kg and saline-treated controls. * *p* < 0.05, when compared to SHR + saline group. Values are mean ± SEM., *n* = 5 for each group.

We investigated whether pretreatment of SHR rats with Fr 2 SySFV by gavage for fifteen days at doses of 50 mg/kg and 100 mg/kg could affect the levels of serum lipid peroxidation as a measure of oxidative stress. Serum malondialdehyde levels were compared in animals pre-treated with Fr 2 SySFV for 15 days p.o. and saline-treated controls (Figure 4). Pre-treatment with Fr 2 SySFV was able to significantly decrease lipid peroxidation (0.9250 nmol/L ± 0.1750 *n* = 4 vs. 2.180 nmol/L ± 0.3891 *n* = 5) at the 100 mg/kg dose level but not at 50 mg/kg. This in vivo antioxidant effect confirms that the phenolic compounds present in the wine fraction are bioavailable when administered by gavage for 15 days and effectively contribute to plasma total antioxidant capacity.

### 3.5. Fr 2 Sy SFV Induces Endothelium-Independent Vasorelaxation in Isolated Rat Superior Mesenteric Rings

**Figure 5:**
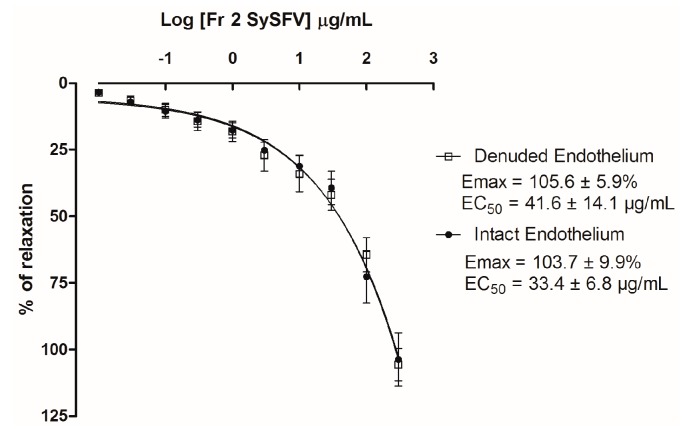
Concentration–response curves showing the relaxant effect induced by Fr 2 SySFV (0.01–1000 μg/mL) in rat mesenteric artery rings pre-contracted with phenylephrine (10 μM) in presence (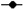) and absence (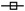) of functional endothelium. Results are expressed as mean ± SEM; *n* = 7 for rings with intact endothelium and *n* = 8 for rings with denuded endothelium.

Red wine flavonoids are known to exert direct vasorelaxant activity. Thus, in order to investigate whether a direct decrease in peripheral resistance was contributing to the hypotensive effect of Fr 2 Sy SFV observed in spontaneously hypertensive rats, we tested in vitro the effect of the fraction on contractions induced by phenylephrine (PHE) on isolated rat superior mesenteric rings. In isolated rat mesenteric artery rings with intact endothelium, Fr 2 SySFV (at 0.01–1000 μg/mL) induced a concentration-dependent relaxation on the contractions induced by Phe (10 mM) (maximum relaxation to contractions by Phe 10 mM = 103.5 ± 9.9%, *n* = 7) (Figure 5). After endothelium removal, the vasorelaxant effect elicited by Fr 2 SySFV was not significantly attenuated (Emax = 105.6 ± 5.9% *n* = 6) (Figure 5). These results demonstrate that a decrease in peripheral resistance is probably involved in the hypotensive effects of Fr 2 SySFV observed in SHR and that the vasorelaxant effect of the fraction is not mediated by endothelium.

## 4. Discussion

Our results demonstrate that the fractionation of the red wine (Syrah variety) from the São Francisco Valley (SFV), yielded a fraction with high total phenolic content. In this work as in a previous study published by our group [4] the highest phenolic content as well as the highest antioxidant activity was found in the neutral (pH 7) acetate fraction (Fr 2 SySFV). According to Menkovic et al., (2014) [32] that used the same fractionation scheme as ours to study a red wine produced in Serbia from the Prokupac variety, the highest levels of phenolics were also observed in the EtOAc fraction obtained at pH 7.0. However, the value of total phenolics found in the study by Menkovic et al. for this fraction was almost half of what we found here for the Syrah red wine from the SFV, confirming the particularly high phenolic content of the red wines from the region. The climate conditions of the SFV and viticulture techniques including the use of controlled-stress irrigation, can contribute to the phenolic profile of these wines as demonstrated previously [33,34]. Our data also confirmed the correlation between the content of phenolic components and the radical scavenging activity of wine and its fractions which has been described in numerous studies [35,36,37,38], despite some authors reporting a lack of correlation between these variables and stressing the importance of the individual phenolic compounds in determining antioxidant activity [39].

The levels of *trans*-resveratrol and quercetin in Fr 2 Sy SFV were similar to those found in the literature for red wines [40,41,42], although higher levels of quercetin and trans-resveratrol have been reported in wines from warm climates such as in the SFV [43,44]. The concentration of *trans*-resveratrol is usually determined by the levels of biotic and abiotic stress in the grapes [45]. However, photoisomerization of *trans*-resveratrol into *cis*-resveratrol is known to occur with high sunlight exposure or during fermentation and storage [46,47,48]. Indeed, levels of cis-resveratrol up to five times those of trans-resveratrol were found in red wines from the SFV [4]. The fractionation of the wine sample proved to be efficient in concentrating bioactive phenolics, since the presence of expected chemical constituents in Fr 2 Sy SFV was confirmed by the experiments using UPLC/MS [26]. Ghiselli et al. (1998) [26] also reported the presence of some procyanidins, catechin, epicatechin, and quercetin-3-glucoside in the EtOAc extract at pH 7.0.

Evidence suggests that hypertensive animals have high levels of oxidative stress [6] and that free radical production can directly or indirectly play a major role in cellular processes implicated in cardiovascular diseases (CVD). Phenolic compounds have been largely considered dietary antioxidant compounds although much controversy still exists about their bioavailability [49]. In the present study the oral administration of Fr 2 Sy SFV in rats SHR for 15 days decreased arterial blood pressure at all doses tested and decreased heart rate at the highest dose (100 mg/kg). In addition, the treatment of animals with the fraction decreased oxidative stress as measured by serum malondialdehyde levels. These results are in line with previous studies, indicating that the antioxidant activity of phenolic compounds such as quercetin [31], vanillic acid [50], and rutin [51] are mainly responsible for the effects of these compounds on blood pressure in different models of hypertension. Recently, quercetin, one of the constituents of Fr 2 SySFV was shown to exert a reduction on blood pressure and on serum malondialdehyde levels in spontaneously hypertensive rats [52]. The antihypertensive effect of Fr 2 Sy SFV in reducing the mean arterial blood pressure is almost equipotent to that of the lyophilized Cabernet Sauvignon red wine from SFV as reported by Ribeiro et al., 2016 [53]. These authors demonstrated that the oral treatment of animals with 100 mg/kg lyophilized wine for 9 days reduced mean arterial blood pressure of chronically L-NAME-treated hypertensive Wistar rats by 29 mmHg compared to a reduction of 24 mmHg in our study. One of the hallmarks of the hypertensive pathophysiological process is endothelium dysfunction provoked by excessive reactive oxygen species production and vascular inflammation [54]. Although numerous studies indicate that red wine and polyphenols present in wine induce an endothelium-dependent vasorelaxant effects [55,56,57], our results have shown that Fr 2 SySFV at concentrations of 0.01–1000 µg/mL induced concentration-dependent relaxation of isolated rat superior mesenteric rings pre-contracted with phenylephrine, an effect which was not attenuated by removal of ring endothelium. In a previous study with a lyophilized red wine from SFV, Luciano et al. (2011) [25] demonstrated the vasorelaxant activity of the wine was significantly attenuated by endothelium removal. It seems plausible to hypothesize that in our work, the fractionation of red wine concentrated phenolic compounds with endothelium-independent vasorelaxant activity. Indeed, it was previously shown that resveratrol is able to induce endothelium-independent relaxation in human internal mammary artery [58]. Quercetin, a flavonoid abundant in most red wines has also been shown to produce endothelium-independent relaxation in arteries of resistance and conductance in rabbits [59].

Considering the 50 mg/kg dose in rats used in this study, Fr 2 SySFV corresponds to an intake of approximately one 750-mL bottle of wine in humans (for a person with an average weight of 70 kg). It is thus not very practical to consider this intake in terms of wine servings, although Fr 2 SySFV could easily be formulated into a dietary supplement or alcohol-free drink. Although we may contemplate vinification practices that could produce wines with chemical composition approaching that of Fr 2 SySFV, the wines would definitely have very different sensorial properties that could impact negatively on their acceptance and commercial viability. Thus, a dietary supplement would definitely be the most viable alternative to achieve an intake of phenolics in human diet that corresponds to the doses administered in this study.

## 5. Conclusions

Taken together our results demonstrate that it is possible to concentrate bioactive and bioavailable phenolics from red wine with important antioxidant and hypotensive activities that are not dependent on intact endothelium function. These results warrant further studies on the effects of individual phenolics on hypertension and on vascular reactivity. Of particular interest would be the identification of the individual phenolics responsible for the endothelium-independent vasorelaxation and hypotensive effects observed for the fraction.

## Figures and Tables

**Table 1:** Total phenolic content of the wine fractions (expressed as gallic acid equivalents, GAE/100 mg fraction) and their antioxidant radical scavenging activity (as EC_50_ values). DPPH: 1,1-diphenyl-2-picrylhydrazyl radical; ABTS: 2,20-azino-bis-3-ethylbenzothiazoline-6-sulphonic acid.

Samples	Total Phenolic Content (mg GAE/100 mg)	EC_50_ (µg/mL)	
		DPPH	ABTS
Fr 1 SySFV	5.57 ± 0.01	56.27 ± 5.50	90.48 ± 1.34
Fr 2 SySFV	58.45 ± 0.01	3.4 ± 0.03	4.65 ± 0.04
Fr 3 SySFV	26.29 ± 0.03	13.25 ± 0.07	11.47 ± 0.55
Ascorbic acid	-	4.38 ± 0.07	-
Trolox	-	-	3.77 ± 0.02

**Table 2:** Identification of compounds present in the fraction Fr 2 SySFV by UPLC-QTOF/MS ^E^.

* Peak	Retention Time (min)	λ_max_	Compounds	Molecular Formula	[M−H]^−^	Fragments (*m/z*)	Calc. Mass	Error (ppm)
1	4.15	278	Catechin	C_15_H_14_O_6_	289.0706	245.0816	289.0712	2.10
2	4.48	278	Procyanidin dimer ^a^	C_30_H_26_O_12_	577.1352	407.0798, 305.0674	577.1352	1.04
3	4.48	278	Procyanidin dimer ^a^	C_30_H_26_O_12_	577.1331	407.0758, 289.0720	577.1352	2.60
4	4.50	322	Caffeic acid	C_9_H_8_O_4_	179.0346	160.8423, 135.0452	179.0344	1.11
5	4.71	278	Epicatechin	C_15_H_14_O_6_	289.0710	245.0824	289.0712	0.72
6	4.98	282	Procyanidin dimer monoglycoside	C_36_H_36_O_17_	739.1848	577.1340, 455.1034	739.1879	>10
7	5.14	285	Myricetin hexoside	C_21_H_20_O_13_	479.0822	316.0234	479.0825	0.63
8	5.21	285	Dihydroquercetin hexoside	C_21_H_22_O_12_	465.1012	319.0827, 301.0351	465.1033	4.51
9	5.31	308	*p*-Coumaric acid	C_9_H_8_O_3_	163.0399	119.0505	163.0395	2.45
10	5.56	272	Syringic acid	C_9_H_10_O_5_	197.0453	160.8495	197.0450	1.52
11	5.56	374	Myricetin	C_15_H_10_O_8_	317.0301	259.0278	317.0303	1.26
12	5.70	ND	Epigallocatechin-coumaroyl ^a^	C_24_H_20_O_9_	451.1026	341.0581, 255.8171	451.1035	0.66
13	5.71	357	Quercertin hexoside	C_21_H_20_O_12_	463.0852	300.0280, 271.0253	463.0876	5.20
14	5.73	357	Myricetin methyl ether hexoside	C_22_H_22_O_13_	493.0988	449.1082, 333.0980	493.0988	1.22
15	5.75	286	Dihydrokaempferol hexoside	C_21_H_22_O_11_	449.1085	285.0404, 229.1086	449.1085	0.22
16	6.29	283	Epigallocatechin-coumaroyl ^a^	C_24_H_20_O_9_	451.1018	341.0667, 271.0651	451.1035	2.43
17	6.34	358	Myricetin dimethyl ether hexoside	C_23_H_24_O_13_	507.1122	477.1027, 341.1033	417.1114	3.35
18	6.44	282	Dihydrokaempferol-rhamnoside	C_21_H_22_O_10_	433.1139	353.1249, 267.1602	433.1140	0.92
19	6.90	305	Epigallocatechin-cinnamoyl	C_24_H_20_O_8_	435.1063	341.0666, 285.0812	435.1085	3.90
20	7.63	371	Quercetin	C_15_H_10_O_7_	301.0345	273.0420, 197.8082	301.0348	1.33
21	7.71	374	Myricetin methyl ether	C_16_H_12_O_8_	331.0448	301.0353, 197.8083	331.0459	3.02
22	8.21	324	Dimethoxy-cinnamic acid	C_11_H_12_O_4_	207.0671	161,0255, 130.0462	207.0663	8.24
23	8.61	360	Luteolin	C_15_H_10_O_6_	285.0404	239.9008, 197.8085	285.0399	1.40
24	8.86	360	Quercetin methyl ether	C_16_H_12_O_7_	315.0510	300.0280, 197.8084	315.0510	1.58
25	9.41	309	Methyl methoxycinnamate	C_11_H_12_O_3_	191.0715	174.9576, 145.0302	191.0714	3.66

* Peak number as marked in the chromatogram shown in Figure 1; ND—representative fragments were not detected; ^a^ positional isomers.
